# Alberta family integrated care^TM^: a review of a family-integrated care model in neonatal intensive care

**DOI:** 10.3389/fped.2026.1915085

**Published:** 2026-08-11

**Authors:** Su Wang, Yuan Wang, Xi Kang, Jie Fu, Liwen Ding, Hong Zhou, Yiyong Fu

**Affiliations:** Neonatal Intensive Care Unit, Chengdu Women’s and Children’s Central Hospital, School of Medicine, University of Electronic Science and Technology of China, Chengdu, China

**Keywords:** Alberta family integrated care, family-centered care, implementation science, narrative review, neonatal intensive care unit, preterm infant

## Abstract

Alberta Family Integrated Care^TM^ (AB-FICare^TM^) is a standardized, theory-driven model that integrates parents as primary caregivers in neonatal intensive care units (NICUs); however, its clinical effectiveness, implementation determinants, and cross-setting applicability require systematic synthesis. This narrative review aims to evaluate the evidence on AB-FICare^TM^ in Level II NICUs, focusing on neonatal and parental outcomes, implementation barriers and facilitators, and transferability to healthcare systems with distinct organizational and cultural contexts. Following SANRA guidelines, we systematically searched PubMed, Embase, Web of Science, and the Cochrane Library for studies published between January 2020 and June 2026. Ten reports from a single Canadian cluster randomized controlled trial met the inclusion criteria. The findings demonstrate that AB-FICare^TM^ significantly reduced the adjusted length of stay by 2.55 days (95% CI: −4.44 to −0.66, *P* = 0.02) without increasing readmissions or emergency visits, and improved parental experiences, specifically enhancing trust, engagement, and discharge readiness. However, exclusive human milk feeding at 2 months was lower in the AB-FICare^TM^ group (aOR = 0.51, 95% CI: 0.31–0.83, *P* = 0.01). Neurodevelopmental outcomes were mixed; while there was a signal for reduced communication delay at 6–24 months, this was not replicated at 18 months, whereas maternal parenting stress consistently predicted developmental delay (aORs > 1.03, *P* < 0.01). Key implementation facilitators included receptive organizational culture and stakeholder engagement, whereas barriers encompassed training burden and competing institutional priorities. Based on these findings, we propose that AB-FICare^TM^ implementation should move from one-size-fits-all adoption toward context-sensitive adaptation, integrating robust lactation support, post-discharge mental health services, and system-level coordination into a comprehensive framework covering NICU stay through community-based follow-up. Future research should prioritize long-term neurodevelopmental follow-up, formal cost-effectiveness analyses adhering to CHEERS standards, and multi-site validation across diverse healthcare settings to establish the generalizability and sustainability of AB-FICare^TM^ benefits beyond the Canadian context.

## Introduction

Neonatal intensive care units (NICUs) have traditionally been environments dominated by advanced medical technology and clinical interventions, with limited integration of families into the care process. This conventional approach often fosters physical and emotional separation between parents and their newborns, particularly preterm or critically ill infants, thereby exacerbating parental anxiety and potentially compromising infant neurodevelopmental outcomes. Globally, an estimated one in ten infants is born preterm, many of whom require specialized care in Level II NICUs marked by a high degree of technological complexity that may prove overwhelming for families (1). Limited parental involvement within the traditional model not only increases parental stress but also impedes the development of parental confidence and caregiving skills essential for the infant's transition from hospital to home. In recognition of these challenges, a paradigm shift has occurred toward models emphasizing the active participation of parents as primary caregivers in the NICU. Family Integrated Care (FICare) represents a broad care philosophy that integrates parents as active members of the neonatal care team, with implementation strategies varying across different countries and NICU Levels (2, 3). These models aim to reduce parent-infant separation, enhance engagement in daily care activities, and foster collaborative decision-making with healthcare providers.

Among these approaches, Alberta Family Integrated Care (AB-FICare^TM^) has emerged as a distinctive, standardized implementation model originating in Alberta, Canada, with well-defined theoretical foundations and structured components (1). AB-FICare^TM^ is grounded in attachment theory, neurodevelopmental care principles, and family systems theory (3). While FICare serves as a broad philosophical framework implemented with varying strategies across different settings, AB-FICare^TM^ represents a distinct, theoretically driven model with three standardized core components: Relational Communication (based on family systems theory), Parent Education (grounded in adult learning and self-efficacy theories), and Parent Support (informed by stress and coping theory) (1, 3, 4). By positioning parents as equal partners and primary caregivers, AB-FICare^TM^ aims to shift the NICU care paradigm from a provider-centric to a family-centric environment, thereby promoting infant neurobehavioral development, reducing medical complications, and enhancing family functioning. Studies evaluating AB-FICare^TM^ have demonstrated promising outcomes. The foundational cluster randomized controlled trial (cRCT) of AB-FICare^TM^ in 10 Level II NICUs reported that AB-FICare^TM^ significantly reduced infant length of stay by 2.55 days after adjustment for site geographic area and infant risk factors (95% CI: −4.44 to −0.66, *P* = 0.02) without concomitant increases in readmissions or emergency department visits (5). Qualitative sub-studies of the same cRCT have revealed that mothers participating in AB-FICare^TM^ experienced enhanced reciprocal trust with healthcare providers, greater involvement in care, and an accelerated “journeying to home” (1), while fathers attributed their positive NICU experience and post-discharge confidence to effective communication and bedside education received during hospitalization (3). Longitudinal follow-up studies of the AB-FICare^TM^ cRCT cohort have suggested a possible protective effect against communication delays at 6–24 months corrected age (6), although no association was found between AB-FICare^TM^ and risk of developmental delay at 18 months corrected age (7).

In addition to the AB-FICare^TM^-specific evidence, the broader FICare literature—encompassing implementations in other countries and NICU Levels—has reported variable outcomes. An international multicentre FICare trial in Level III NICUs demonstrated improved weight gain and reduced maternal stress yet failed to show a significant reduction in length of stay (8); conversely, a Chinese FICare cRCT reported both a shorter length of stay and higher breastfeeding rates (9). These differences highlight the importance of differentiating the specific AB-FICare^TM^ model from other FICare implementations, as findings from one context may not be directly generalizable to another.

Despite these promising outcomes, the widespread adoption and standardization of AB-FICare^TM^ face several challenges. Implementation studies have demonstrated that organizational readiness, resource availability, staff engagement, and cultural context significantly influence the success of integrating this model into routine NICU practice (5). Barriers such as limited physical space for parental presence, staffing constraints, and infection control concerns have been reported (5, 10). Healthcare providers' attitudes and perceptions toward family integration vary, necessitating tailored education and training programs to foster a supportive climate for AB-FICare^TM^ (5, 10). Cultural and socioeconomic diversity further complicate implementation, underscoring the need for adaptable strategies that respect local values and family dynamics while maintaining fidelity to the core principles of the model. This is especially critical in settings with restrictive visitation policies or limited infrastructure. The COVID-19 pandemic has also highlighted vulnerabilities in family-centered approaches, where visitation restrictions exacerbated parent-infant separation, thereby emphasizing the importance of innovative solutions such as digital health tools to sustain family engagement. These challenges underscore the need for systematic implementation strategies tailored to local contexts.

As neonatal care evolves and NICU capacity rapidly expands in healthcare settings with distinct organizational structures and cultural norms, such as those in China, this review aims to synthesize evidence from studies directly evaluating the AB-FICare^TM^ model in Level II NICUs, while drawing on broader FICare literature for context. Furthermore, it seeks to identify factors influencing the adaptation and implementation of models similar to AB-FICare^TM^ in resource-constrained and culturally diverse healthcare systems. We critically analyze the model's theoretical underpinnings, implementation strategies, clinical effectiveness, and barriers and facilitators to adoption, and future directions to offer guidance for clinicians and policymakers seeking to advance family-integrated care practices.

## Methods

### Review design and rationale

We conducted a narrative review to synthesize the existing evidence on the theoretical underpinnings, real-world implementation outcomes, barriers, and facilitators of the standardized Alberta Family Integrated Care (AB-FICare^TM^) model in neonatal intensive care units (NICUs), and to identify knowledge gaps and priorities for future clinical and translational research.

A narrative synthesis approach was selected over a systematic review with meta-analysis for two primary reasons. First, the included literature was anticipated to exhibit substantial heterogeneity in study designs (ranging from randomized controlled trials to qualitative interviews), intervention delivery protocols, and outcome measurement instruments, which would preclude meaningful quantitative pooling. Second, our objective extended beyond effect estimation to include a comprehensive examination of implementation contexts, stakeholder perspectives, and contextual modifiers—dimensions that are more appropriately captured through narrative interpretation than through statistical aggregation.

This review was reported in accordance with the Scale for the Assessment of Narrative Review Articles (SANRA) guidelines, a six-item quality assessment tool specifically developed for narrative reviews in biomedical literature (11, 24). The review protocol was not prospectively registered, given the narrative and exploratory nature of this work.

### Search strategy

We systematically searched four electronic biomedical databases—PubMed, Embase, Web of Science Core Collection, and the Cochrane Library—to identify relevant peer-reviewed literature. The final searches were executed on July 1, 2026, by two independent information specialists with expertise in neonatal and family-centered care research. The search timeframe was restricted to publications from January 1, 2020, through June 30, 2026, to capture the most current evidence following the formal dissemination of the AB-FICare^TM^ framework.

The core Boolean search strategy was designed to capture studies specifically evaluating the AB-FICare^TM^ model in Level II NICUs, while also identifying relevant broader FICare literature for contextual purposes. To achieve this, the strategy combined controlled vocabulary terms (MeSH in PubMed; EMTREE in Embase) and free-text keywords across four conceptual domains: (1) the intervention or care model (Family Integrated Care OR FICare OR Alberta Family Integrated Care OR AB-FICare OR family centered care OR family centred care); (2) the target population [preterm infant OR premature infant OR prematurity OR Infant, Premature (MeSH)]; (3) the setting [Neonatal Intensive Care Unit OR NICU OR neonatal unit OR Intensive Care Units, Neonatal (MeSH)]; and (4) outcomes or thematic topics (clinical outcome OR parental mental health OR parental stress OR parental anxiety OR parental self-efficacy OR implementation barrier OR implementation facilitator OR staff perception OR breastfeeding OR length of stay OR family integration).

The complete, database-specific search strings—including field tags, filters, and syntax adaptations for Embase, Web of Science, and the Cochrane Library—are provided in Supplementary Table S1 to ensure full reproducibility. In brief, Embase searches employed.ti,ab. fields and/exp EMTREE terms with equivalent Boolean logic across the same four conceptual domains; Web of Science utilized TS = (topic search) for title, abstract, and keyword fields; and the Cochrane Library was searched via the standard advanced search interface using MeSH-derived and keyword-based terms. To maximize sensitivity and capture the full range of study designs—including randomized controlled trials, cohort studies, qualitative research, and mixed-methods evaluations—no additional filters for study design were applied during the electronic search.

### Inclusion and exclusion criteria

Studies were considered eligible if they met all of the following inclusion criteria:

Study design: Empirical original research, including randomized controlled trials, quasi-experimental studies, prospective or retrospective cohort studies, cross-sectional surveys, and qualitative interview studies that evaluated the standardized AB-FICare^TM^ practice model in Level II or Level III NICUs;
Study population: Preterm infants (gestational age < 37 weeks) and/or their parents/caregivers, with at least one measurable outcome reported—neonatal outcomes (e.g., weight gain velocity, length of hospital stay, breastfeeding rates or breast milk feeding rates, readmission rate, nosocomial infection rate) or parental outcomes (e.g., parental stress, anxiety, depression, satisfaction with care, self-efficacy, or perceived family integration);
Publication type: Peer-reviewed, full-text, original research articles published in English between January 2020 and June 2026;
Data availability: Studies must provide complete, extractable primary quantitative or qualitative data.

Studies were excluded if they met any of the following criteria:

Non-English publications, conference abstracts, editorials, commentaries, letters, or opinion pieces without accessible full-text manuscripts or primary empirical data;
Studies on generic family-centered care models that did not specifically operationalize or evaluate the AB-FICare^TM^ framework;
Animal experiments, *in vitro* studies, case reports (*n* < 5), or case series without a concurrent comparison group;
Duplicate publications derived from the same research dataset (in such cases, only the most recent and complete version were retained);
Studies conducted in Level I NICUs or non-tertiary neonatal settings without standardized AB-FICare^TM^ protocol implementation.

### Study selection and data extraction

**Figure 1:**
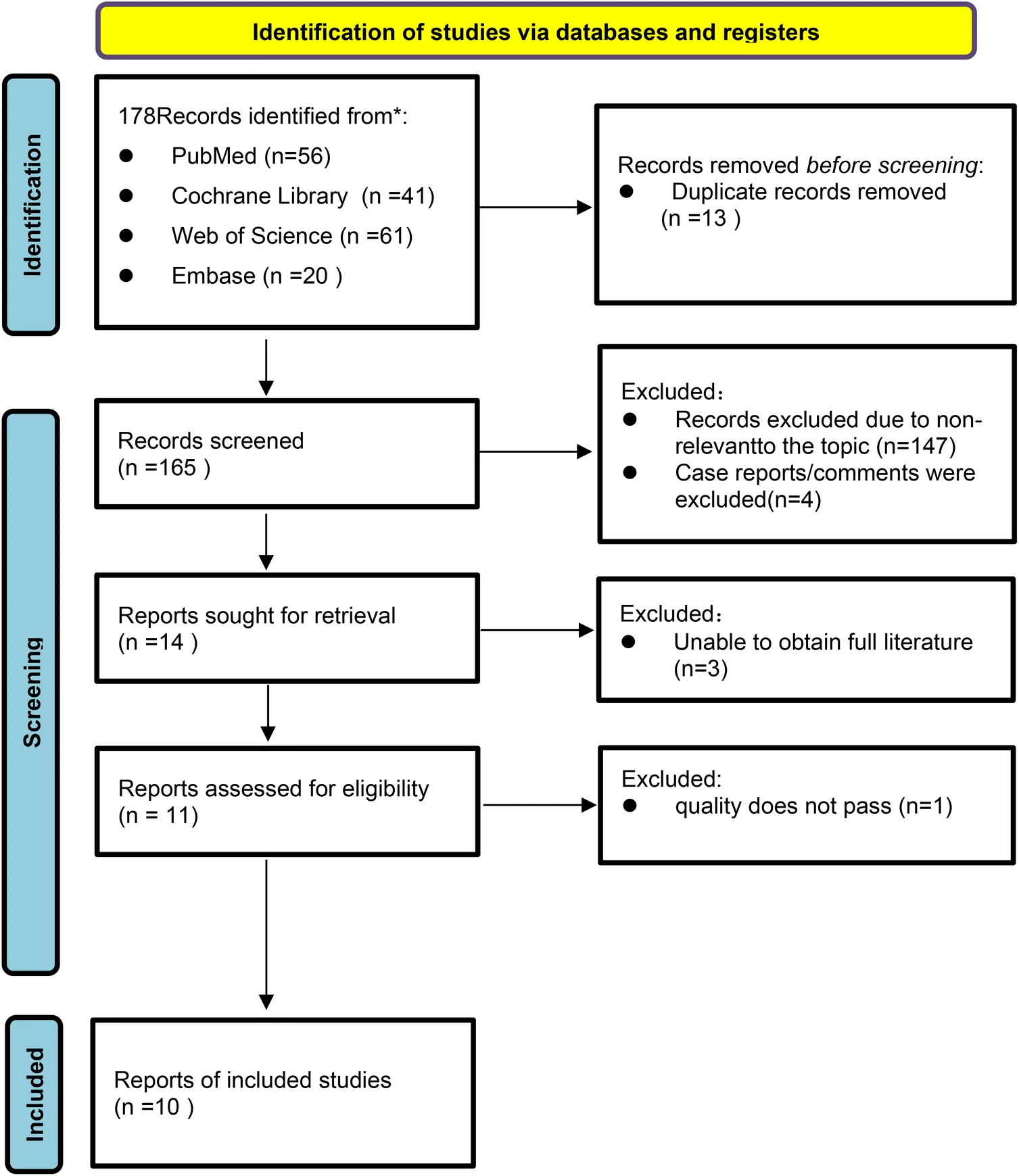
PRISMA 2020 flow diagram of the study selection process. A total of 178 records were identified from four databases. After duplicate removal and screening, 10 studies met the inclusion criteria and were included in the narrative synthesis. AB-FICare^TM^, Alberta Family Integrated Care; NICU, neonatal intensive care unit. Adapted from Page et al. (13).

Retrieved citations were exported to EndNote X9 for deduplication. Two independent reviewers screened titles/abstracts and then full texts using a pilot-tested form. Disagreements were resolved by consensus with a third senior neonatology expert. Inter-reviewer agreement was high (*κ* = 0.89 for title/abstract screening; *κ* = 0.93 for full-text selection). The screening process is shown in the PRISMA-ScR flow diagram (Figure 1). After deduplication, 165 unique records were identified; following two rounds of screening, 10 studies met the inclusion criteria.

From each included study, we extracted: study design; country; patient population; sample size; intervention characteristics; main outcomes; principal findings. Data extraction was performed independently by the same two reviewers, with cross-verification to minimize errors. Table 1 summarizes these key characteristics of all included studies.

**Table 1:** Characteristics of included studies on AB-FICare^TM^ in NICUs.

Study ID (Author, Year)	Country	Study design	NICU level	Patient population	Sample size	Intervention characteristics	Main outcomes	Principal findings	MMAT score	Quality Level
Wilson JM et al., (10)	Canada	Concurrent mixed-methods sub-study (cluster RCT quantitative + thematic qualitative analysis)	II	Moderate/late preterm infants (32^0^/^7^–34^6^/^7^ weeks gestation); their mothers	326 mothers (FICare: 194; SC: 132)	Standardized Alberta FICare^TM^: daily ≥6 h maternal bedside stay, free parking pass, provider relational communication + parent education online modules; Control = standard family-centered routine care without AB-FICare standardized training/tools	Primary: Direct non-medical out-of-pocket expenditures (OOPE: parking, food, lodging, childcare, travel etc.); Secondary: Qualitative experiential factors driving high/low spending	Total non-medical OOPE showed no statistically significant difference between AB-FICare and standard care groups (*U* = 12,679.50, *P* = 0.882). AB-FICare mothers spent less on parking (*P* < 0.001) but more on food and lodging (*P* = 0.014, *P* < 0.001). Two core qualitative themes: transportation barriers, residential proximity to NICU determine extreme spending; Caesarean delivery, long-distance residence increased family financial burden.	3	Moderate
Moe et al., (6)	Canada	Prospective longitudinal follow-up of cRCT	II	Moderate/late preterm infants (32^0^/^7^–34^6^/^7^ weeks GA) and their mothers	Study 1: 330 mothers, 387 infants (FICare: 223; SC: 164); Study 2: 50 mothers, 61 infants (FICare: 30; SC: 31)	Alberta FICare^TM^: theoretically driven psychoeducational model; three components: relational communication, parent education, parent support; parents encouraged ≥6 h/day in NICU	Primary: Infant developmental delay risk (ASQ-3 communication, problem-solving, personal-social domains). Secondary: Maternal depression/anxiety (EPDS, STAI), parenting stress (PSI-4-SF); parent-child interaction quality (PCITS, only Study 2).	Study 1 (2 mo CA): no effect on any ASQ domain. Study 2 (6–24 mo CA): lower risk of communication delay in FICare group vs. SC (*P* = 0.014, 95% CI 0.01–0.62); no effect on problem-solving or personal-social.	3	Moderate
Benzies et al., (7)	Canada	Prospective longitudinal follow-up of cluster RCT	II	Moderate/late preterm infants (32^0^/^7^–34^6^/^7^ weeks GA) and their mothers	257 mothers, 298 infants (FICare: 111 mothers, 135 infants; SC: 146 mothers, 163 infants)	Alberta FICare^TM^: psychoeducational model; three components: relational communication, parent education, parent support; parents encouraged to be present ≥6 h/day in NICU	ASQ-3 (global development); ASQ:SE-2 (social-emotional); BITSEA (behavioral); maternal psychosocial distress (CESD-R, STAI, PSI-4-SF, GSE)	No association between FICare and risk of developmental delay at 18 months CA. Higher maternal parenting stress (PSI) was associated with increased risk of developmental delay on all measures (ASQ-3: aOR = 1.046, *P* < 0.01; ASQ:SE-2: aOR = 1.061, *P* < 0.01; BITSEA: aOR = 1.033, *P* < 0.01).	3	Moderate
Dien et al., (1)	Canada	Qualitative study using interpretive description informed by grounded theory methods	II	Mothers of moderate/late preterm infants (32^0^/^7^–34^6^/^7^ weeks GA) from 10 Level II NICUs	26 mothers (AB-FICare^TM^: 14; SC: 12)	Alberta FICare^TM^: three components: Relational Communication, Parent Education, Parent Support; parents encouraged ≥6 h/day in NICU; mothers received parking pass	Mothers’ experiences of parenting in NICU; major theme “Journeying to Home” with six categories: Recovering from Birth, Adapting to NICU, Caring for Baby, Coping with Daily Disruption, Seeing Progress, Supporting Parenting	Major theme “Journeying to Home” identified. AB-FICare^TM^ mothers experienced enhanced reciprocal trust with HCPs, more involvement in care, participation in bedside rounds, and accelerated journey to home compared to SC.	5	High
Ringham et al., (14)	Canada	Institutional ethnography	II	Mothers of moderate/late preterm infants (32^0^/^7^–34^6^/^7^ weeks GA) from Alberta FICare^TM^ intervention sites	101 mothers (all from AB-FICare^TM^ group)	Alberta FICare^TM^: three components: relational communication, parent education, parent support; parents encouraged ≥6 h/day in NICU; parent journals provided for daily recording	Mothers’ work of being present in NICU; three discourses identified: feeding policies and practices, roller coaster of emotions, mothering work	Mothers’ work coordinated by institutional processes prioritizing discharge progression (feeding, weight gain) over emotional support. Tensions identified: lack of decision-making power in feeding, difficulty navigating NICU rules, managing competing demands. Journal format organized maternal knowledge around feeding metrics and weight gain.	5	High
Benzies et al., (4)	Canada	Cluster randomized controlled trial (cRCT)	II	Moderate/late preterm infants (32^0^/^7^–34^6^/^7^ weeks GA) and their mothers; recruited from 10 Level II NICUs in Alberta, Canada	Final sample: 353 infants, 308 mothers (Alberta FICare^TM^: 308 mothers, 353 infants; SC: 306 mothers, 365 infants). Enrolled: 654 mothers, 765 infants (Alberta FICare^TM^: 325 mothers, 375 infants; SC: 329 mothers, 390 infants)	Alberta FICare^TM^: theoretically driven, psychoeducational model with 3 components: Relational Communication (family systems theory), Parent Education (adult learning + self-efficacy theories), Parent Support (stress and coping theory); parents encouraged ≥6 h/day in NICU; mothers received parking pass; multimodal education (pathway, bedside teaching, group sessions, app)	Primary: Infant hospital LOS. Secondary: Hospital readmissions to 2 mo CA; ED visits to 2 mo CA; feeding type at discharge; maternal psychosocial distress (STAI, EPDS, PSS:NICU); maternal parenting self-efficacy (PMP S-E)	Adjusted for site geographic area and infant risk factors, Alberta FICare^TM^ significantly reduced infant LOS by 2.55 days (95% CI: −4.44 to −0.66, *P* = 0.02; permutation test *P* = 0.05). Unadjusted difference (−1.96 days, *P* = 0.22) was not significant. No significant differences in secondary outcomes: readmissions (6.2% vs. 6.6%, *P* = 0.85); ED visits (23.5% vs. 25.5%, *P* = 0.54); feeding type (*P* = 0.57); maternal psychosocial distress or parenting self-efficacy at discharge (all *P* > 0.05).	5	High
Murphy et al., (15)	Canada	Cluster randomized controlled trial (cRCT)	II	Moderate/late preterm infants (32^0^/^7^–34^6^/^7^ weeks GA) and their mothers; recruited from 10 Level II NICUs in Alberta, Canada	Final sample: 353 infants, 308 mothers (Alberta FICare^TM^: 308 mothers, 353 infants; SC: 306 mothers, 365 infants); Enrolled: 654 mothers, 765 infants	Alberta FICare^TM^: theoretically driven, psychoeducational model with 3 components: relational communication, parent education, parent support; parents encouraged ≥6 h/day in NICU; mothers received parking pass; multimodal education	Primary: Proportion of infants who regained BW by 14 days of life. Secondary: Apnea of prematurity; duration of TPN; time to full enteral feeds; time to first skin-to-skin; time to first breastfeed; time to regain BW; proportion who regained BW at discharge	No difference in proportion of infants who regained BW by 14 days between groups (70.4% FICare vs. 66.0% SC, *P* = 0.381). Regional site subgroup: significantly more FICare infants regained BW by 14 days (89.1% vs. 67.0%, *P* = 0.01). FICare group had earlier skin-to-skin (0.7 vs. 1.1 days, *P* < 0.001), shorter TPN duration (4.1 vs. 4.7 days,*P* = 0.02), earlier full enteral feeds (4.1 vs. 4.6 days, *P* = 0.03). Fewer FICare infants regained BW at discharge (70.5% vs. 78.0%, *P* = 0.02)—attributed to shorter LOS (infants discharged before regaining BW).	5	High
Zanoni et al., (5)	Canada	Qualitative process evaluation substudy using CFIR	II	Healthcare providers and hospital administrators involved in the AB-FICare^TM^ cRCT	32 participants (AB-FICare: 16; SC: 16)	Alberta FICare^TM^: three components: relational communication, parent education, parent support; parents encouraged ≥6 h/day in NICU; train-the-trainer model	Barriers and facilitators to AB-FICare^TM^ implementation from HCP and administrator perspectives using CFIR framework	Key facilitators: receptive implementation climate, intervention compatibility with existing practices, available resources and access to knowledge, engagement of key stakeholders, positive participant outcomes, reflecting/evaluating progress. Key barriers: design quality/packaging (overwhelming initial information), relative priority (competing initiatives), learning climate. Swing factors: intervention source, cost, peer pressure, external policy, staff needs/resources, structural characteristics, organisational incentives, knowledge/beliefs.	5	High
Shafey et al., (3)	Canada	Qualitative substudy of cRCT (interviews + journal analysis)	II	Fathers of moderate/late preterm infants (32^0^/^7^–34^6^/^7^ weeks GA) from 10 Level II NICUs	Interviews: 13 fathers (FICare: 9; SC: 4); Journals: 24 fathers (all from FICare group)	Alberta FICare^TM^: three components: relational communication, parent education, parent support; parents encouraged ≥6 h/day in NICU; mothers received parking pass	Fathers’ experiences in NICU; seven themes: fear of the unknown, mental preparation, identifying fathers’ role, parenting with supervision, effective communication, post-NICU transition, family life	Fathers in FICare group attributed positive NICU experience and post-discharge confidence to effective communication, bedside attention, and education received. FICare fathers felt more involved, bonded earlier, and better prepared for discharge than SC fathers. SC fathers described delayed attachment until post-discharge.	5	High
Moe et al., (16)	Canada	Prospective longitudinal follow-up of cRCT (2-month follow-up)	II	Moderate/late preterm infants (32^0^/^7^–34^6^/^7^ weeks GA) and their mothers	455 infants, 381 mothers (FICare: 204 infants, 175 mothers; SC: 251 infants, 206 mothers)	Alberta FICare^TM^: psychoeducational model; three components: relational communication, parent education, parent support; parents encouraged ≥6 h/day in NICU	Human milk feeding at age 2 months (3 categories: EHM, Non-EHM, NHM)	Compared to SC, FICare mothers less likely to provide EHM vs. NHM (aOR = 0.51, 95% CI: 0.31–0.83, *P* = 0.01). No group difference between EHM and Non-EHM (aOR = 1.04, *P* = 0.85). EHM at discharge (aOR = 4.16–8.74, *P* < 0.001), higher BSES-SF (aOR = 1.04–1.09, *P* < 0.001), higher education (college: aOR = 2.22, *P* < 0.004), and employment/maternity leave (aOR = 2.63–1.95, *P* < 0.001) were associated with EHM at 2 months.	4	High

AB-FICare^TM^, Alberta family integrated care; NICU, neonatal intensive care unit; RCT, randomized controlled trial; cRCT, cluster randomized controlled trial; SC, standard care; LOS, length of stay; OOPE, out-of-pocket expenses; EHM, exclusive human milk; NHM, no human milk; HCP, healthcare providers; MMAT, mixed methods appraisal tool (2018 version; total score range: 0–5).

MMAT Quality Level Definitions: High (score 4–5): indicates a low risk of bias, allowing findings to be presented with greater confidence; Moderate (score 3): suggests some methodological limitations, requiring findings to be interpreted with moderate caution.

### Methodological quality

We appraised the methodological quality of all included studies using the 2018 Mixed Methods Appraisal Tool (MMAT), a validated instrument designed for the critical appraisal of quantitative, qualitative, and mixed-methods research. Two reviewers independently scored each study (0–5), with disagreements resolved by consensus. No study was excluded based on score; rather, MMAT ratings were used to stratify evidence into high (4–5), moderate (3), and low (0–2) quality tiers to calibrate interpretive confidence (12). All MMAT scores are reported in Table 1.

Owing to heterogeneity in study designs, intervention protocols, and outcome measures, a formal meta-analysis was not performed. Quantitative outcomes were summarized descriptively (ranges/medians), with effect sizes reported verbatim from original sources. Qualitative and implementation findings are reported narratively, with key findings extracted from source texts through group consensus. All synthesized findings were interpreted with consideration of MMAT quality scores, with consideration also given to potential publication bias. Quantitative data are traceable to Table 1.

### Study characteristics summary

To facilitate critical appraisal of the evidence base, we developed a comprehensive summary table (Table 1) that details, for each included study, the country of origin, study design, NICU Level, patient population and sample size, intervention characteristics, main outcomes, principal findings, and MMAT quality score. This table enables readers to independently assess the composition, scope, and methodological quality of the evidence supporting our narrative synthesis, thereby enhancing the transparency and reproducibility of the review. A narrative summary of the overall evidence base—including the distribution of study designs, geographic origins, sample size ranges, and MMAT score categories—is presented at the beginning of the Results section, accompanied by an explicit cross-reference to Table 1.

## Results

### Study selection and characteristics

A systematic search across four electronic databases (PubMed, Embase, Web of Science Core Collection, and the Cochrane Library) yielded 178 unique citations. After removal of duplicates and two rounds of independent dual-reviewer screening, 10 original empirical studies met the predetermined eligibility criteria and were incorporated into the narrative synthesis. The complete study selection process is displayed in the PRISMA-ScR flow diagram (Figure 1).

All 10 eligible papers were conducted in Alberta's Level II NICUs and derived from the same provincial cluster randomized controlled trial (cRCT) evaluating standardized AB-FICare^TM^ (4). The evidence base falls into four distinct categories:

1. Five quantitative analyses: the core primary cRCT plus three follow-up outcome substudies (4, 6, 7, 15, 16);
2. Three independent qualitative studies exploring maternal, paternal and institutional caregiving experiences (1, 3, 14);
3. One concurrent mixed-methods substudy focusing on families’ non-medical out-of-pocket costs (10);
4. One CFIR-based qualitative process evaluation analyzing implementation barriers and facilitators (5).

All included studies underwent methodological quality assessment via the 2018 MMAT tool, with results summarized in Table 1. Seven articles were rated as high quality (MMAT score ≥ 4), and three were graded as moderate quality (MMAT = 3); no studies of low quality (MMAT ≤ 2) were identified. When interpreting results, higher confidence is assigned to findings from high-quality research, while conclusions from moderate-quality studies are interpreted with caution. All eligible articles were published between 2020 and 2026, consistent with the search timeframe defined in the Methods section.

### Quantitative findings

#### Length of hospital stay & neonatal clinical outcomes

The landmark multicenter cluster RCT enrolling 765 preterm infants and 654 mothers across 10 Canadian Level II NICUs identified significant reductions in adjusted NICU length of stay (LOS) under standardized AB-FICare^TM^. After adjusting for site differences and infant baseline risks, the adjusted mean LOS was 17.62 days for the AB-FICare^TM^ group vs. 20.16 days for standard care, with an adjusted mean difference of −2.55 days (95% CI: −4.44 to −0.66, *P* = 0.02; permutation test *P* = 0.05). The unadjusted intergroup difference of −1.96 days lacked statistical significance (*P* = 0.22) (4).

Secondary growth and feeding indicators were analyzed on the same cRCT cohort (353 AB-FICare^TM^ infants, 365 standard care infants). No overall between-group difference was detected in 14-day birth weight recovery (70.4% vs. 66.0%, *P* = 0.381). Regional subgroup analysis revealed a notable trend: AB-FICare^TM^ infants showed a higher 14-day weight recovery rate (89.1% vs. 67.0%, *P* = 0.01). Infants in the intervention group achieved earlier skin-to-skin contact (0.7 vs. 1.1 days, *P* < 0.001), reduced duration of parenteral nutrition (4.1 vs. 4.7 days, *P* = 0.02), and earlier full enteral feeding (4.1 vs. 4.6 days, *P* = 0.03). A lower proportion of AB-FICare^TM^ infants had recovered birth weight by discharge (70.5% vs. 78.0%, *P* = 0.01); this finding is attributable to their shortened hospital stay (15). These secondary endpoints originated from unadjusted exploratory subgroup analyses without correction for multiple comparisons; their findings should only be regarded as hypothesis-generating rather than definitive clinical evidence.

#### Two-month human milk feeding outcomes

A 2-month corrected age follow-up (455 infants, 381 mothers) assessed breastfeeding outcomes within the same trial cohort. Compared with mothers receiving standard care, caregivers in the AB-FICare^TM^ group had lower odds of providing exclusive human milk (EHM) vs. no human milk feeding (aOR = 0.51, 95% CI: 0.31–0.83, *P* = 0.01). No significant difference was observed in the odds of EHM vs. partial human milk feeding (aOR = 1.04, *P* = 0.85). Independent positive predictors of sustained EHM at 2 months included exclusive breastfeeding status at discharge, higher BSES-SF self-efficacy scores, maternal college education, and stable maternity leave arrangements (16).

#### Long-term neurodevelopmental follow-Up

An 18-month corrected age follow-up (298 mothers, 298 infants) assessed global, social-emotional, and behavioral developmental risks via ASQ-3, ASQ:SE-2, and BITSEA. No independent association was observed between AB-FICare^TM^ exposure and developmental delay across all three scales (all aOR included the null value, *P* > 0.01). By contrast, elevated maternal parenting stress consistently predicted higher delay risk on every assessment tool (all aOR > 1.03, *P* < 0.01) (7).

A second developmental follow-up stratified the analysis by corrected age groups: 2 months and 6–24 months. At the 2-month window (330 mothers, 387 infants), AB-FICare^TM^ produced no measurable impact on ASQ-3 communication, problem-solving or personal-social domains. At 6–24 months (50 mothers, 61 infants), infants in the model group had a significantly lower risk of communication delay (*P* = 0.014, 95% CI: 0.01–0.62). This result is interpreted cautiously due to the very small subsample size and wide confidence interval approaching the null value; replication in larger independent cohorts is required to validate the signal (6).

#### Out-of-pocket non-medical expenditures

A mixed-methods substudy nested within the core cRCT enrolled 326 mothers to compare household non-medical costs. AB-FICare^TM^ families spent substantially less on parking (median $0 vs. $119, *P* < 0.001) due to the provision of complimentary hospital parking permits, yet incurred higher food (median $291 vs. $235, *P* = 0.014) and lodging costs (median $150 vs. $10, *P* < 0.001). This cost substitution resulted in no statistically significant difference in total expenditure between groups (*U* = 12,679.50, *P* = 0.882). The qualitative thematic analysis identified two core drivers of extreme financial burden: long travel distances and residential distance to the NICU (10).

### Qualitative findings

Three independent qualitative substudies nested within the Alberta FICare^TM^ multicenter cRCT (4) employed complementary analytical approaches to explore the lived experiences of mothers and fathers and the institutional routines that shape parental participation in Level II NICUs.

#### Mothers' experiences

A qualitative study employing an interpretive descriptive approach informed by grounded theory enrolled 26 mothers and identified the overarching thematic construct Journeying to Home, consisting of six interconnected subcategories: Recovering from Birth, Adapting to the NICU, Caring for Baby, Coping with Daily Disruption, Seeing Progress, and Supporting Parenting (1). Compared with mothers receiving standard care, participants receiving AB-FICare reported higher Levels of mutual trust with clinical staff, more active engagement in their infant's daily care, consistent attendance at multidisciplinary bedside rounds, and a smoother, more empowering discharge preparation trajectory (MMAT = 5/5) (1).

#### Fathers' experiences

By combining semi-structured interviews with 13 fathers and a thematic analysis of 24 paternal journal entries, this substudy achieved data triangulation and identified seven core experiential themes: fear of the unknown, mental preparation, paternal role identification, supervised infant care, effective clinical communication, post-NICU transition, and family life balance (3). Fathers in the AB-FICare group attributed their positive experience during hospitalization and sustained post-discharge parenting confidence to targeted bedside education and individualized clinician communication. In contrast, fathers receiving standard care commonly described delayed parent-infant attachment until after discharge, whereas fathers in the AB-FICare group reported earlier bonding and greater readiness for the transition home (MMAT = 5/5); these qualitative findings are detailed elsewhere (3).

#### Institutional processes

An institutional ethnography drawing on 101 maternal care journals examined how hospital organizational priorities structured maternal caregiving workloads (14). Institutional workflows prioritized infant feeding metrics and weight gain targets over maternal emotional support, giving rise to three persistent tensions: limited autonomy over infant feeding decisions, the burdensome nature of navigating NICU rules, and conflict between hospital and domestic care responsibilities. The standardized parent journal functioned as a ruling text that directed maternal attention toward quantifiable growth indicators, thereby reinforcing hospital operational priorities (MMAT = 5/5; 14).

#### Brief synthesis of qualitative evidence

Collectively, these three high-quality qualitative studies indicate that AB-FICare strengthens parent-clinician partnerships and facilitates early attachment among both mothers and fathers. However, institutional performance metrics prioritizing weight gain and feeding progression generate an unaddressed emotional burden for mothers, even within the standardized intervention model.

### Implementation barriers and facilitators

A CFIR-based qualitative substudy involving 32 clinical and administrative stakeholders explored core enablers and barriers to AB-FICare^TM^ implementation (5). Six consistent implementation facilitators were identified: receptive ward culture, alignment with existing workflows, adequate training resources, cross-hierarchy stakeholder engagement, positive infant/parent outcomes, and regular iterative progress reviews. Three major obstacles included the excessive volume of initial training materials, low institutional priority amid competing quality projects, and a suboptimal learning climate. Multiple context-dependent moderators—including intervention cost, staff shortages, external policies, institutional incentives, and provider beliefs—also influenced site-specific implementation fidelity (MMAT = 5/5) (5).

## Discussion

### Summary of evidence

This narrative review synthesizes evidence from 10 studies that specifically assessed the Alberta Family Integrated Care (AB-FICare^TM^) model in Level II NICUs. The evidence base included one foundational cluster randomized controlled trial (cRCT) (4), its secondary and longitudinal follow-up analyses (6, 7, 15, 16), qualitative sub-studies (1, 3, 14), a mixed-methods sub-study (10), and one implementation process evaluation (5).

The strongest evidence indicates that AB-FICare^TM^ significantly reduces infant length of stay in Level II NICUs, with an adjusted reduction of 2.55 days (95% CI: −4.44 to −0.66, *P* = 0.02) without associated increases in readmissions or emergency department visits (4).

### Comparison with broader FICare literature

To contextualize the AB-FICare^TM^-specific findings, it is important to compare them with evidence from the broader FICare literature—implementations of FICare in other countries and NICU Levels—while maintaining a clear distinction between the two evidence streams.

The international multicentre FICare cRCT in Level III NICUs (*n* = 1,786) (17) reported improved weight gain trajectories and reduced maternal stress and anxiety but did not demonstrate a significant reduction in length of stay, which was evaluated as a secondary resource-use outcome rather than a primary endpoint—a key difference from the AB-FICare^TM^ cRCT, which found a significant adjusted reduction of 2.55 days (95% CI: −4.44 to −0.66, *P* = 0.02) in Level II NICUs (4).

A Chinese FICare cRCT (*n* = 601) (9) reported shorter length of stay and higher breastfeeding rates in the FICare group, findings that align with the AB-FICare^TM^ evidence for length of stay but contrast with the human milk feeding findings at 2 months (16).

These comparisons highlight the importance of distinguishing between AB-FICare^TM^-specific evidence and broader FICare literature. Readers should exercise caution against overgeneralizing findings from non-Alberta FICare studies to the AB-FICare^TM^ model.

### Interpretation of findings

#### Reduction in length of stay

A 2.55-day reduction in length of stay (LOS) associated with AB-FICare^TM^ (4) is clinically meaningful. For health systems, this translates to reduced bed occupancy and potential cost savings; for families, earlier discharge may reduce parent-infant separation and overall financial burden, although these benefits are partially offset by significantly higher expenditures on food (median $291 vs. $235, *P* = 0.014) and lodging (median $150 vs. $10, *P* < 0.001) (10). The lack of an increase in readmissions or emergency department visits suggests that the earlier discharge was not achieved at the expense of patient safety (4).

Enhanced parental confidence and competence, as reported in qualitative studies (1, 3), combined with structured parent education (4), may have accelerated discharge readiness. However, as maternal psychosocial distress and self-efficacy did not differ between groups at discharge (4), the underlying mechanism likely involves increased parental competence and the earlier attainment of feeding milestones rather than improvements in maternal mental health alone.

#### Mixed developmental outcomes

The contrasting developmental findings observed in the 6–24 month follow-up (6) compared with the larger 18-month study (7) may be attributed to differences in sample size (61 vs. 298 infants), measurement tools, or diminishing protective effects over time. In the 6–24 month study, AB-FICare^TM^ was associated with a lower risk of communication delay (*P* = 0.014, 95% CI: 0.01–0.62), whereas the 18-month study reported no significant association with developmental delay on any measure (all adjusted odds ratios included the null value, *P* > 0.05) (7).

Crucially, the consistent association between higher maternal parenting stress and increased developmental delay risk across all measures in the 18-month cohort (7) suggests that the post-discharge family environment may exert a greater influence than NICU-based interventions for long-term development. This aligns with Bronfenbrenner's bioecological theory (18), which positions the family microsystem as central to child development. Interventions targeting post-discharge parenting stress—potentially through home visitation programs or parenting support groups—may be critical for optimizing developmental outcomes in moderate and late preterm infants.

#### Human milk feeding

The counterintuitive finding that AB-FICare^TM^ mothers were less likely to provide exclusive human milk (EHM) at 2 months (16) —with an adjusted odds ratio of 0.51 (95% CI: 0.31–0.83, *P* = 0.01) for EHM vs. no human milk—may reflect earlier discharge, which may reduce the time available to establish breastfeeding in the supportive NICU environment, or competing demands introduced by earlier transition home. Breastfeeding intention was not measured, raising the possibility that AB-FICare^TM^ mothers had similar intentions but faced greater post-discharge barriers.

The strong association between breastfeeding self-efficacy (BSES-SF) at discharge and EHM at 2 months (16)—each one-point increase in BSES-SF was associated with a 4%–9% increase in the odds of EHM—suggests that enhancing maternal confidence during the NICU stay may be more impactful than the care model alone. Integrating individualized lactation consultation, peer support, and post-discharge follow-up into the AB-FICare^TM^ framework represents a key practical priority.

#### Implementation considerations

The CFIR-based process evaluation (5) identified site-specific critical determinants—staff resources, structural characteristics, and organisational incentives—that influenced AB-FICare^TM^ adoption, indicating that one-size-fits-all implementation strategies are unlikely to succeed. The train-the-trainer model yielded variable fidelity, with fidelity audits showing 71% adherence at intervention sites but only 30% compliance at standard care sites (4), highlighting the necessity of ongoing monitoring and support. Future implementations should embed AB-FICare^TM^ training into new hire orientation and incorporate annual refresher training to maintain fidelity over time.

#### Global applicability to healthcare settings with distinct organizational structures and cultural norms

While derived from a single high-income integrated health system, the AB-FICare^TM^ findings provide transferable insights to healthcare settings with distinct organizational structures and cultural norms, particularly in the context of expanding NICU capacity in China (9). The successful adaptation of this model requires careful consideration of the following factors:

1. System-level coordination: Healthcare settings with distinct organizational structures and cultural norms within fragmented healthcare systems may need to strengthen system-level coordination before implementing complex behavioral interventions like AB-FICare^TM^. Implementation science frameworks, such as the CFIR employed in the AB-FICare^TM^ process evaluation (5), can facilitate systematic adaptation planning.
2. Cultural adaptation of relational communication: In cultures with strong hierarchical medical traditions, healthcare providers may initially resist sharing decision-making with parents (19). Training programs emphasizing the evidence base for parental involvement and providing practical communication tools may help shift provider attitudes over time (25).
3. Robust breastfeeding support: The unexpected observation that mothers in the AB-FICare^TM^ group were less likely to provide EHM at 2 months (16) is particularly relevant for healthcare settings with distinct organizational structures and cultural norms. In these settings, where breastfeeding rates vary widely, earlier discharge may disproportionately impede the establishment of breastfeeding. It is recommended that lactation consultation, peer support, and post-discharge follow-up be integrated into any adaptation of the AB-FICare^TM^ model.
4. Post-discharge family support: The consistent association between maternal parenting stress and developmental delay (7) suggests that post-discharge family support is as critical as NICU-based interventions. Healthcare settings characterized by distinct organizational structures and cultural norms, especially where community-based health services are limited, should consider investing in home visitation programs, primary care-based parenting support, or mobile health interventions to sustain the benefits of family-integrated care beyond discharge (26).

In summary, while AB-FICare^TM^ demonstrates potential as a model of family-integrated care, its transferability to healthcare settings with distinct organizational structures and cultural norms necessitates a systematic consideration of workforce, cultural, economic, and policy contexts. The Chinese FICare trial (9) offers proof-of-concept evidence that FICare can be successfully adapted, but ongoing evaluation and context-sensitive implementation are critical to ensuring sustainable impact.

### Implications for practice and policy

#### For clinicians and NICU teams

The findings support the adoption of structured, theoretically driven family-integration models like AB-FICare^TM^ to reduce length of stay and enhance parental experiences. The relational communication component emerges as a critical factor for building reciprocal trust with families—a finding consistently reported across qualitative studies (1, 3). Training programs should emphasize communication skills, and bedside rounds should actively encourage parental participation. However, clinicians should recognize that earlier discharge may not be universally beneficial; families facing transportation barriers, geographic distance from the NICU, or limited social support may require additional resources to navigate the transition home (10).

#### For health system administrators and policymakers

Evidence indicates that AB-FICare^TM^ can be implemented effectively in Level II NICUs within an integrated health system. However, the intervention is resource-intensive, requiring staff training, physical space modifications, and concrete family support measures such as parking passes. The CFIR-based process evaluation identified site-specific critical determinants—staff needs and resources, structural characteristics, and organisational incentives—that should inform implementation planning (5). The train-the-trainer model resulted in varying levels of fidelity [71% at intervention sites vs. 30% at standard care sites, (4)], underscoring the need for ongoing fidelity monitoring and booster training sessions.

The finding that maternal parenting stress—rather than the NICU care model—was consistently associated with developmental outcomes at 18 months (7) has policy implications. Post-discharge maternal mental health services, home visitation programs, and community-based parenting support appear to be equally critical to NICU-based interventions for long-term child development. Health systems should consider integrating post-discharge support into the AB-FICare^TM^ framework.

#### For researchers

The mixed evidence on developmental outcomes and human milk feeding highlights several knowledge gaps. A major limitation of current evidence is that all included studies are derived from a single Canadian provincial cohort with homogeneous participants; therefore, cross-national multi-center validation across diverse populations and healthcare systems is urgently needed to expand generalizability. Future research should prioritize:

Longer-term follow-up beyond 18 months to assess neurodevelopmental trajectories through school age
Mediation analyses to identify mechanisms underlying the length-of-stay reduction and the unexpected human milk feeding findings
Implementation science studies to examine how AB-FICare^TM^ can be adapted for culturally diverse populations and resource-limited settings
Cost-effectiveness analyses comparing AB-FICare^TM^ with standard care, accounting for both health system savings and family out-of-pocket expenditures
Father-specific interventions, given that fathers in this review reported positive experiences but also described perceiving themselves as secondary parents (3)

From a health economics perspective, existing evidence is limited to a single mixed-methods substudy assessing exclusively family out-of-pocket costs, with an absence of comprehensive cost-utility, cost-effectiveness, and cost-benefit modeling from hospital, healthcare payer, and societal viewpoints. We establish standardized health economic evaluation as a top research priority for subsequent AB-FICare trials, mandating adherence to the 2022 Consolidated Health Economic Evaluation Reporting Standards (CHEERS) framework to systematically quantify bed occupancy savings, gains from reduced long-term readmissions, and cumulative household financial burdens (28). Rigorous economic modeling will provide actionable cost data for hospital administrators and policymakers to inform the allocation of budgets for sustained implementation.

### Challenges during implementation and strategies to overcome them

Implementing AB-FICare^TM^ in NICUs presents multifaceted challenges stemming from cultural, organizational, and individual factors, necessitating targeted strategies to facilitate effective implementation and long-term sustainability. These challenges can be categorized into two main groups: universal challenges—common across most NICU settings—and context-specific challenges that are particularly salient in healthcare settings characterized by distinct organizational structures and cultural norms, such as those in China, where NICU capacity is rapidly expanding but systemic and cultural conditions differ markedly from the Canadian context in which AB-FICare^TM^ was initially developed and validated (4, 5).

#### Universal challenges

A common barrier across all settings is resistance among healthcare providers to the shift away from traditional roles, particularly concerns about parental errors in infant care and the potential impact on clinical outcomes. This apprehension reflects existing medical authority models and highlights the need for ongoing education and cultural change initiatives that emphasize the safety and benefits of parental involvement (4). Another universal challenge is the socioeconomic and occupational constraints faced by parents, which limit their ability to sustain a continuous presence in the NICU. Work obligations, financial pressures, and transportation difficulties can hinder parental participation, thereby undermining the model's effectiveness (10). In the Alberta context, flexible visitation policies—including extended and night-time access—and tangible supports such as parking passes have been implemented to address these barriers (4). The establishment of parent mentor programs, in which experienced parents provide peer support and guidance, has also been shown to enhance parental engagement and alleviate anxiety, fostering a supportive community within the NICU (5). Additionally, skepticism regarding the cost-effectiveness of AB-FICare^TM^ persists among healthcare administrators, despite evidence indicating reductions in length of stay and healthcare expenditures associated with the model (4). Conducting comprehensive cost-benefit analyses and disseminating positive economic data are critical to securing institutional buy-in and resource allocation.

#### Context-specific challenges in healthcare settings characterized by varying organizational structures and cultural norms

In addition to universal challenges, scaling AB-FICare^TM^-like models within healthcare settings with distinct organizational structures and cultural norms—particularly in China (20, 21), a country bearing the world's largest preterm birth burden and possessing rapidly expanding NICU capacity—faces systemic and cultural barriers that necessitate context-specific solutions.

Staffing constraints and workforce models. In many Chinese NICUs, nurse-to-patient ratios indicate a significantly heavier workload than in Canadian Level II NICUs, with nurses often managing 6–8 infants per shift compared to 2–3 in Alberta (22). This high workload allows little time for the relational communication and individualized parent education that are core components of AB-FICare^TM^ (3, 4). Moreover, the physician staffing model differs: while Alberta Level II NICUs are typically staffed by neonatologists or hospital-based paediatricians, many Chinese NICUs rely on community paediatricians who rotate through multiple facilities, which reduces continuity of care and the feasibility of consistent multidisciplinary bedside rounds with parental participation (23). Addressing these workforce constraints may require task-shifting strategies, such as training dedicated parent educators or family mentors to deliver education and support, thereby alleviating the burden on bedside nurses (5, 27).

Parental presence policies and cultural norms. The requirement for parental presence in the NICU for at least 6 h per day—a core component of AB-FICare^TM^ (1)—presents implementation challenges in settings where cultural norms or hospital policies restrict parental access. In some Chinese NICUs, traditional practices have historically limited parental presence, where parents were often excluded from the NICU or permitted only during designated visiting hours (9). While the Chinese FICare trial successfully challenged these norms and demonstrated that parents could be effectively integrated into care (9), implementing this model required substantial cultural change, including staff education and the reconfiguration of physical spaces to accommodate parents. Hospital policies in some settings may also restrict overnight stays or require parents to rotate between multiple caregivers, undermining the consistency of parental involvement that AB-FICare^TM^ emphasizes (1).

Socioeconomic and geographic disparities. China's vast geographic and socioeconomic diversity poses additional challenges. Families from rural areas may face long travel distances to tertiary or regional NICUs, limiting their ability to sustain daily visitation (9). The financial burden of transportation, accommodation, and food—similar to findings in the Alberta context (10)—may be disproportionately greater for families in middle-income countries, where out-of-pocket healthcare expenditures constitute a larger proportion of household income. These disparities necessitate targeted financial support mechanisms, such as subsidized accommodation near hospitals, transportation vouchers, and flexible visitation policies that support families from distant locations.

Cultural barriers to paternal involvement. Cultural barriers also pose challenges, particularly in contexts where paternal involvement is traditionally limited or where language and religious differences affect family participation. In many Chinese families, fathers are often expected to prioritize work outside the home, thereby limiting their presence in the NICU (1). Tailored interventions, such as providing multilingual educational materials, culturally sensitive care practices, and father-specific support programs, are important for overcoming these obstacles (3). Studies indicate that addressing these multifactorial challenges through collaborative, context-specific strategies enhances the feasibility and acceptability of AB-FICare^TM^, promoting equitable and effective family integration in diverse NICU settings (5).

#### Strategies to overcome context-specific barriers

Drawing on insights from both the AB-FICare^TM^ implementation experience (5) and the Chinese FICare trial (9), several strategies may facilitate adaptation in healthcare settings characterized by distinct organizational structures and cultural norms:

Phased Implementation and Cultural Adaptation: Rather than immediate full-scale adoption of the AB-FICare^TM^ model, healthcare facilities with distinct organizational structures and cultural norms may benefit from a phased approach that prioritizes culturally acceptable components—such as parent education and kangaroo mother care—before introducing more resource-intensive elements such as family mentors or daily multidisciplinary rounds with full parental participation (9).

Workforce innovation and task-shifting. Training dedicated parent educators, family mentors, or community health workers to deliver parent education and psychosocial support could mitigate the workload pressures on overstretched nursing staff while preserving fidelity to AB-FICare^TM^ components (5).

Technology-enhanced parental engagement. In settings where the physical presence of parents is constrained by distance or work obligations, telehealth tools—such as video monitoring, mobile applications for parent education, and virtual participation in bedside rounds—can extend parental involvement beyond the NICU (6). Multiple real-world digital parenting interventions implemented in Chinese tertiary women's and children's hospitals offer actionable insights for adapting AB-FICare to local contexts: WeChat mini-programs dedicated to preterm lactation guidance, AI neonatal nursing chatbots, and 24-hour video consultation hotlines have been widely deployed in Beijing, Guangzhou, and Sichuan provincial NICUs (29–31). Longitudinal trials conducted within China have confirmed that six months of continuous remote coaching via mobile platforms effectively alleviates parental anxiety and improves sustained exclusive breastfeeding rates among rural and commuting families with limited daily bedside attendance (32). Such digital modules can be seamlessly integrated into localized AB-FICare adaptation packages to mitigate staffing shortages and restrictive visiting policies.

Financial support mechanisms. Government-subsidized accommodation, transportation vouchers, and meal support for NICU families could mitigate the economic burden of parental presence, particularly for families from low-income or rural backgrounds (10).

Advocating for Policies Supporting Parental Presence. Health system leaders and policymakers across diverse healthcare settings characterized by varying organizational structures and cultural norms should consider revising hospital visitation policies to facilitate unrestricted 24-hour parental access, providing the physical infrastructure (e.g., parent lounges, sleeping accommodations) to support sustained parental presence, and establishing national or provincial guidelines for family-integrated care tailored to local contexts (9).

### Limitations

Several methodological limitations warrant consideration when interpreting these findings.

First, the evidence base is geographically and methodologically narrow—all ten included studies are drawn from a single cluster randomized controlled trial conducted within Alberta's integrated health system. This inherently limits the generalizability of these findings to other healthcare systems, higher-level NICUs, or more diverse patient populations. Notably, the 18-month follow-up retained only 39.3% of the original cohort, with participants disproportionately partnered, Caucasian, and higher-income, raising concerns about selection bias. This demographic homogeneity also extended to the qualitative substudies.

Second, while thematically consistent, the qualitative evidence was derived from a predominantly homogeneous sample, limiting its transferability to Indigenous, immigrant, low-income, or single-parent families.

Third, health economic evidence remains incomplete; the sole mixed-methods substudy analyzed only direct out-of-pocket costs, lacking formal cost-effectiveness or cost-utility modelling. Consequently, the net financial benefit and health-system sustainability of AB-FICare^TM^ remain unclear.

Identified in this study as priorities for future research, important knowledge gaps persist beyond these methodological constraints—particularly regarding neurodevelopmental trajectories beyond 18 months, the role of digital health tools in sustaining parental engagement, and scalable implementation strategies for healthcare settings with diverse organizational structures and cultural norms.

### Strengths

Despite these limitations, this review has several strengths. First, we conducted a systematic search of four major databases and explicitly differentiated between AB-FICare^TM^-specific evidence and broader FICare studies, addressing a key methodological concern identified in the literature. Second, we employed the Mixed Methods Appraisal Tool (MMAT) to appraise methodological quality and leveraged quality ratings to calibrate interpretive confidence, ensuring that conclusions are commensurate with the strength of the underlying evidence. Third, by anchoring the synthesis within established theoretical frameworks—attachment theory, neurodevelopmental care principles, and family systems theory—and encompassing a comprehensive range of study designs (quantitative, qualitative, and mixed-methods), the review moves beyond simple descriptive aggregation to offer mechanistically informed, multi-dimensional interpretations of heterogeneous outcomes, particularly regarding the mixed developmental and breastfeeding findings. Fourth, the inclusion of implementation process evaluations provides insights into barriers and facilitators that can inform scale-up efforts (5). Finally, the review was conducted in accordance with SANRA guidelines, ensuring transparent reporting (24).

## Conclusions

This narrative review indicates that AB-FICare^TM^ is associated with a clinically significant reduction in infant length of stay in Level II NICUs (adjusted reduction: 2.55 days) and consistent improvements in parental experiences across multiple qualitative domains. However, studies on long-term neurodevelopmental and breastfeeding outcomes have yielded inconsistent results, and the current evidence base is limited to a single Canadian provincial cohort, thereby restricting direct generalizability to other healthcare systems. Implementation must be context-sensitive, addressing site-specific barriers in staffing and infrastructure. Beyond these structural considerations, the inconsistent breastfeeding outcomes underscore the need to integrate robust lactation support and post-discharge follow-up into locally adapted implementation strategies. Most critically, the strong association between maternal parenting stress and developmental delay suggests that post-discharge family support—including mental health services—may be as essential as NICU-based interventions. In healthcare settings with distinct organizational structures and cultural norms—such as those in China—systematic adaptation that addresses workforce constraints, cultural expectations regarding family presence, and socioeconomic diversity is essential. Future research should prioritize long-term follow-up, formal cost-effectiveness analyses, and multi-site validation to determine whether the benefits of AB-FICare^TM^ can be effectively and equitably replicated across diverse global healthcare systems, particularly in resource-limited settings.
