# Supplementary material for: Alberta family integrated careTM: a review of a family-integrated care model in neonatal intensive care
Source: Front Pediatr. 2026 Aug 11;14:1915085. doi: 10.3389/fped.2026.1915085 (PMC13504315; doi:10.3389/fped.2026.1915085)
Supplement: Supplementary file 1 [file Table1.docx]

# **Supplementary Table S1. Complete Search Strategies for Each Database**

| **Database** | **Search Date** | **Search String** |
| --- | --- | --- |
| PubMed | July 1, 2026 | (  (  "Family Integrated Care"[Title/Abstract] OR  "FICare"[Title/Abstract] OR  "Alberta Family Integrated Care"[Title/Abstract] OR  "AB-FICare"[Title/Abstract] OR  "family centered care"[Title/Abstract] OR  "family centred care"[Title/Abstract]  )  AND  (  "preterm infant"[Title/Abstract] OR  "premature infant"[Title/Abstract] OR  "prematurity"[Title/Abstract] OR  "Infant, Premature"[MeSH Terms]  )  AND  (  "Neonatal Intensive Care Unit"[Title/Abstract] OR  "NICU"[Title/Abstract] OR  "neonatal unit"[Title/Abstract] OR  "Intensive Care Units, Neonatal"[MeSH Terms]  )  AND  (  "clinical outcome"[Title/Abstract] OR  "parental mental health"[Title/Abstract] OR  "parental stress"[Title/Abstract] OR  "parental anxiety"[Title/Abstract] OR  "parental self-efficacy"[Title/Abstract] OR  "implementation barrier"[Title/Abstract] OR  "implementation facilitator"[Title/Abstract] OR  "staff perception"[Title/Abstract] OR  "breastfeeding"[Title/Abstract] OR  "length of stay"[Title/Abstract] OR  "family integration"[Title/Abstract]  )  AND "Humans"[Filter]  AND "English"[Filter]  AND ("2020/01/01"[Date - Publication] : "2026/06/30"[Date - Publication]) ) |
| Embase | July 1, 2026 | (  (  'family integrated care':ti,ab OR  'ficare':ti,ab OR  'alberta family integrated care':ti,ab OR  'ab-ficare':ti,ab OR  'family centered care':ti,ab OR  'family centred care':ti,ab  )  AND  (  'preterm infant':ti,ab OR  'premature infant':ti,ab OR  'prematurity':ti,ab OR  'prematurity'/exp  )  AND  (  'neonatal intensive care unit':ti,ab OR  'nicu':ti,ab OR  'neonatal unit':ti,ab OR  'intensive care units, neonatal'/exp  )  AND  (  'clinical outcome':ti,ab OR  'parental mental health':ti,ab OR  'parental stress':ti,ab OR  'parental anxiety':ti,ab OR  'parental self-efficacy':ti,ab OR  'implementation barrier':ti,ab OR  'implementation facilitator':ti,ab OR  'staff perception':ti,ab OR  'breastfeeding':ti,ab OR  'length of stay':ti,ab OR  'family integration':ti,ab  )  )  Limits: humans/lim; english/lim; 2020-2026/py |
| Web of Science Core Collection | July 1, 2026 | TS=("Family Integrated Care" OR "FICare" OR "Alberta Family Integrated Care" OR "AB-FICare" OR "family centered care" OR "family centred care")  AND TS=("preterm infant" OR "premature infant" OR "prematurity")  AND TS=("Neonatal Intensive Care Unit" OR "NICU" OR "neonatal unit")  AND TS=("clinical outcome" OR "parental mental health" OR "parental stress" OR "parental anxiety" OR "parental self-efficacy" OR "implementation barrier" OR "implementation facilitator" OR "staff perception" OR "breastfeeding" OR "length of stay" OR "family integration") Limits: English; 2020-2026 |
| Cochrane Library | July 1, 2026 | (  (  "Family Integrated Care":ti,ab,kw OR  "FICare":ti,ab,kw OR  "Alberta Family Integrated Care":ti,ab,kw OR  "AB-FICare":ti,ab,kw OR  "family centered care":ti,ab,kw OR  "family centred care":ti,ab,kw  )  AND  (  "preterm infant":ti,ab,kw OR  "premature infant":ti,ab,kw OR  "prematurity":ti,ab,kw OR  "Infant, Premature"/exp  )  AND  (  "Neonatal Intensive Care Unit":ti,ab,kw OR  "NICU":ti,ab,kw OR  "neonatal unit":ti,ab,kw OR  "Intensive Care Units, Neonatal"/exp  )  AND  (  "clinical outcome":ti,ab,kw OR  "parental mental health":ti,ab,kw OR  "parental stress":ti,ab,kw OR  "parental anxiety":ti,ab,kw OR  "parental self-efficacy":ti,ab,kw OR  "implementation barrier":ti,ab,kw OR  "implementation facilitator":ti,ab,kw OR  "staff perception":ti,ab,kw OR  "breastfeeding":ti,ab,kw OR  "length of stay":ti,ab,kw OR  "family integration":ti,ab,kw  ) )  Limits: Publication Year 2020 to 2026; English |

Table Legend：

Supplementary Table S1 Complete search strings adapted for each database. All searches were executed on July 1, 2026, with the timeframe restricted to publications from January 1, 2020, through June 30, 2026. Language filters were applied as specified. The Boolean logic was translated across platforms to ensure consistent retrieval across the four conceptual domains (intervention/care model; target population; setting; outcomes/topics). PubMed used [Title/Abstract] and [MeSH] field tags; Embase used .ti,ab. fields and /exp EMTREE terms; Web of Science used TS= (topic search); and the Cochrane Library used the standard advanced search interface with keyword‑based terms. No additional filters for study design were applied during the electronic search to maximize sensitivity.
